# Editorial: World mental health day 2022: key drivers of risk to mental health services and innovative solutions

**DOI:** 10.3389/frhs.2024.1456603

**Published:** 2024-07-19

**Authors:** Nelson Shen, Sagar Jilka, Kim Sawchuk

**Affiliations:** ^1^ Digital Mental Health Lab, Krembil Centre for Neuroinformatics, Centre for Addiction and Mental Health, Toronto, ON, Canada; ^2^ Institute for Health Policy, Management and Evaluation, Dalla Lana School of Public Health, University of Toronto, Toronto, ON, Canada; ^3^ Warwick Medical School, University of Warwick, Coventry, United Kingdom; ^4^ Institute of Psychiatry, Psychology, and Neuroscience, Kings College London, London, United Kingdom; ^5^ Aging and Communication Technologies (ACT), Department of Communication Studies, Concordia University, Montreal, QC, Canada

**Keywords:** mental health, health services, innovation, socio-cultural adaptation, biopyschosocial model, COVID-19, human-centred design

**Editorial on the Research Topic** World mental health day 2022: key drivers of risk to mental health services and innovative solutions

## Introduction

The COVID-19 pandemic triggered a global mental health crisis, increasing stress and fueling anxiety and depressive disorders (1). In a world where already one in eight people are living with a mental disorder globally, the pandemic has put more strain on services, skills, and funding available for mental health. The pandemic has also had dire consequences in low and middle-income countries (LMICs), where the treatment gap, that is the number of people who need care actually get the required care, for mental health conditions has expanded (2).

We launched this special topic to coincide with World Mental Health Day 2022, to showcase the latest multidisciplinary research and insights into the global situation of mental health services during and post-pandemic. Our goal was to identify the key drivers of risk to mental health services, and innovations in service delivery modes that improve accessibility or coordination.

There were four strong themes embodied across the submissions received. First there were discussions of the impact of COVID-19 on service utilization and those providing care. The second theme spoke to the importance of attending to contextual differences and acknowledging cultural adaptations to different health services. The third theme focuses on the role of engaging patients and people with lived experience in designing services. The final group of papers underscore the significance of the biopsychosocial model, which sees mental health issues as a part of the larger holistic landscape of health, rather than as a clinical practice separate and apart.

## Impact of COVID-19 pandemic on service utilization and workforce

Our collection highlights the critical need for enhanced mental health support for the public and healthcare workforce during crises like the COVID-19 pandemic. In Van den Broeck et al.'s study, set in Belgium, we saw a significant rise in psychological and anxiety-related issues in out of hours consultations for psychological problems since the onset of the pandemic. These consultations remained elevated even between COVID-19 waves. This increased use of out of hours care for mental health reasons can be an indicator of the unmet mental health needs in a population.

The increased strain on healthcare workers during the pandemic cannot be overlooked as burnout was already a significant threat to the well-being of the workforce prior to the pandemic. We wanted to shine a light on this issue and acknowledge the tremendous work of our healthcare teams during this period. Eder and Meyer's work stresses the need for interventions to address these harmful coping strategies and improve working conditions for long-term care nurses. They reveal that altruistic motivations in nursing can lead to self-endangering behaviors and increased exhaustion. Similarly, Bannon et al. suggest that promoting positive psychological constructs can mitigate burnout. Targeted interventions to build resilience, particularly among younger, female, and patient-facing healthcare workers, are crucial, and they emphasize the need for healthcare systems to create supportive work environments and prioritize mental well-being to reduce burnout rates.

Leadership behavior and health policy changes are essential to create a supportive work environment and prevent burnout. As observed by Hale and Davis, fewer than 14% of medical schools in the United States adhered to the Association of American Medical College's guidelines for mental health service provision. They discuss the need to include these guidelines in accreditation standards to improve adherence, underscoring the importance of policy to enable change.

## Cultural and contextual adaptations to mental health services

Alongside burnout issues, it is important to consider cultural contexts to build capacity and ensure positive mental health. For instance, in low-and-middle-income countries (LMICs), it is well-known that help-seeking for mental illness is pluralistic, and alternative sources of care, such as traditional and faith healers or lay community health workers, are viewed by many as a valued community resource, which may help fill the “treatment gap” in low- and middle-income countries (LMICs). The World Health Organization (WHO) developed the “Mental Health GAP” (mhGAP) course, which aims to upskill and train non-specialist clinical staff in basic diagnosis and treatment. In many settings, psychiatric services are scarce or unaffordable, so initiatives like the mhGAP are critical. This is evidenced in the submission by Kuule et al., who found that providing mental health care in the community—away from a hospital setting, substantially increased the number of people accessing mental health care, and providing training to health center-based staff in mhGAP contributed to this.

Help-seeking for mental illness is dynamic and diverse with common concurrent or sequential use of various forms of treatment in the search for a “cure”. Respectful dialogue and mutual learning not only between lay health workers and biomedical workers, but also caregivers and people with lived experience, can identify shared understandings, as well as opportunities for questioning, discovery, and transformative change. All efforts must address existing power hierarchies and health system challenges—particularly those faced during the pandemic such as virtual working, as well as engage with the meaningful activities, understanding the intrinsic values and needs of people with lived experience and their families. This calls for a public mental health approach, in which such collaborations are embedded within communities and supported by policies and interventions to address social as well as spiritual and medical needs.

## Understanding lived experience for effective innovation

There is a growing recognition that understanding experiences and perspectives is critical in developing, implementing, and adapting interventions and services tailored to the needs of those using it (3), especially as it relates to patients and individuals with lived experience. Adam et al., highlighted the importance of choice and individualized care needs in traditional inpatient treatment and novel inpatient equivalent home treatment settings in Germany (3). Their qualitative study identified choice and care needs as key elements in enhancing patient satisfaction and treatment efficacy. At an organizational and systems level, Yu et al. provides a reflection on the integration of a patient, family, and persons with lived experience lived experience team (PFLE) in shaping the development of the BrainHealth Databank (BHDB), a large data initiative to advance personalized care and research at a large academic mental health hospital in Canada. These accounts include various types of engagement across the spectrum (4), ranging from “consultation” in the development of a research and care coordination portal, “involvement” in co-designing a patient-facing trajectory dashboard, to “partnership” in the BHDB governance.

Understanding the patient experience has become increasingly important with the post-pandemic shift to virtual delivery of care, as seen in two Canadian studies. Pulia et al. used admissions data to understand the feasibility of the rapid deployment Virtual Crisis Stabilization Unit as a safe, effective and feasible mechanism to provide mental health crisis care, particularly during times when traditional service delivery is disrupted. Their findings provide insights on delivering equitable and patient-centred services in Winnipeg, Canada. Henderson et al., report on their community-based participatory research approach in successfully transitioning to a virtual delivery of the Raising the Curtain on Lived Experience of Dementia (RTC) initiative in British Columbia—a creative 5-year community-based, art-engaged project to support older adults who experience isolation, loneliness, financial challenges, and mental illness. The community collectively navigated this transition and demonstrated the potential for virtual delivery to foster a sense of community, empowerment, and well-being, all of which challenges the stigma associated with dementia and the ageist assumptions about technology use.

## Embracing a biopsychosocial mindset to mental health

As with the RTC initiative, several papers in this issue encourage us to think broadly about the complexity of mental health recovery, and the dynamic interpersonal and psychological systems that shape an individual's well-being (5). This dynamic is observed in the comparative study by Padyab et al., where they found differences in the types and severity of stressors experienced by Swedish and Norwegian police officers between 2018 and 2020. Furthermore, stress levels in Sweden decreased over time whereas no change or a slight increase was observed in Norway. They hypothesize that changes in organizational structure and interventions in safety and security may have improved stress for Swedish police officers. The role of societal issues and population dynamics were also explored by Abdelhadi; they found that psychological distress adversely affects satisfaction and experiences with healthcare services of American cancer survivors. Furthermore, those with psychological distress have limited access to mental health service, with many forgoing access due to its affordability. Nakimuli-Mpungu et al. will be exploring the association between psychological interventions to improve treatment outcomes. Their protocol details a pilot randomized controlled trial to evaluate the feasibility, acceptability, and preliminary effectiveness of incorporating group support psychotherapy with antiretroviral therapy for young people living with HIV in Uganda.

This collection also saw many articles highlight the biological social interactions in the complexity of mental illnesses. Ouyang et al. saw dynamic changes in frequency and types of chief complaints to their psychological crisis hotline over the different stages of the pandemic in Jiangsu, China. This study illustrates the interplay between psychological complaints (e.g., anxiety, depression, obsessive compulsive symptoms) and social factors (e.g., quarantine measures, fear or infection), but also highlights the interactions with biological responses (e.g., physical discomfort, insomnia). These findings further the case to take an integrated biopsychosocial approach to healthcare—a concept that is neither novel nor the norm (6). This gap was seen in Tredget et al.'s service evaluation of the London Mental Health Trust in the United Kingdom, where clinical staff, service users, and careers recognized the importance of integrating physical health and community support into the healthcare experience of those with serious mental illness. However, there was variability in how physical healthcare was delivered, calling for the need for guidance and leadership on how to navigate and streamline the complexities of integrated care within the Trust. Lesson can be drawn from Cano-Prieto et al. biopsychosocial approach in their MOSAIC project, which is a social initiative that accounts for psychological well-being and social determinants of health alongside traditional medical care. Their holistic approach to treatment and recovery was meaningful in engaging individuals with severe mental illness in Catalonia (Spain), fostering social inclusion, life satisfaction, resilience, hope, and personal recovery.

## Perspectives

In our special topic collection, we highlight the urgent need for innovative strategies to address the disparities experienced in healthcare and transform mental health services beyond the pandemic. This global body of work reveals a common set of challenges we face, from supporting the current workforce, to requiring capacity for care delivery in underserved areas, to the transition to digital provision of care, to the challenges in providing support tailored to the recovery needs of specific populations. However, the insights from these studies highlight the value of organizational and system leadership, systemic resiliency, cultural contexts, and lived experience as critical pieces in overcoming these challenges.

The insights generated from this collection reinforces common ideas and approaches in the mental health discourse. There is a need to prioritize a holistic multidisciplinary collaborative approach—one in which embraces the complexity of delivering health services, valuing lived experience and cultural context in designing supportive environments for the workforce and the population (7). It is evident that a dynamic, responsive biopsychosocial approach to mental health is essential in enhancing resilience, promoting positive psychological well-being, and empowering individuals to cope and adapt to a variety of complex and dynamic circumstances. We hope readers find this issue useful in mental health service innovation and advocacy. We also hope this issue contributes to the ongoing efforts in shifting the paradigm on how we frame, govern, and deliver mental health services.
